# Lethal Leptospiral Pulmonary Hemorrhage: An Emerging Disease in Buenos Aires, Argentina

**DOI:** 10.3201/eid0809.010499

**Published:** 2002-09

**Authors:** Alfredo Seijo, Héctor Coto, Jorge San Juan, Juan Videla, Bettina Deodato, Beatriz Cernigoi, Oscar García Messina, Oscar Collia, Diana de Bassadoni, Ricardo Schtirbu, Alejandro Olenchuk, Gleyre Dorta de Mazzonelli, Alberto Parma

**Affiliations:** * Hospital F.J. Muñiz, Buenos Aires, Argentina; † Fundación Mundo Sano, Buenos Aires, Argentina; ‡ Hospital P. Piñero, Buenos Aires, Argentina; § Centro de Salud No. 18, Buenos Aires, Argentina; ¶ Universidad La Plata, Provincia de Buenos Aires, Argentina; # Servicio Nacional de Sanidad Animal y Calidad Agroalimentaria, Buenos Aires, Argentina; ** Laboratorio de Biotecnología, Facultad de Ciencias Veterinarias, Universidad Nacional del Centro, Tandil, Buenos Aires, Argentina

**To the Editor:** In the Buenos Aires metropolitan area, 40–100 cases of human leptospirosis are reported annually. Occasional epidemic outbreaks have been characterized by mild leptospiral illness. Severe illness with acute renal failure and extensive cutaneous and visceral hemorrhages (always accompanied by jaundice) has been observed only rarely. A review of our data for 1990–1999 showed that 276 human cases were diagnosed; 43 of these were characterized by pneumonia alone or associated with another syndrome. No severe pulmonary hemorrhage due to leptospirosis was detected in these cases (Table), and the case-fatality rate was <1% (1).

**Table:** Clinical findings in human leptospirosis, Hospital F.J. Muñiz, Buenos Aires, 1990–1999

Year	Cases	J**^a^**	N	M	P	H	IL
1990	130	37	37	26	15	14	19
1991	27	12	9	2	7	7	4
1992	25	16	13	7	7	4	0
1993	29	10	7	0	5	3	10
1994	12	4	3	0	4	1	2
1995	12	4	1	0	0	1	1
1996	7	4	1	1	0	1	0
1997	12	8	5	0	3	0	2
1998	14	6	5	2	1	0	1
1999	8	3	3	1	1	1	1

^a^J, jaundice; N, nephritis; M, meningitis; P, pneumonia; H, hemorrhages; IL, influenza-like

Rodents and dogs are considered major reservoirs for this zoonotic illness*. Rattus norvegicus* (78%) and *R. rattus* (22%) are the most widely distributed and predominant species. Rodent abundance has been estimated by the Hayne’s Index1 as 0.414–0.465. Prevalence of leptospiral infection as measured by kidney culture of captured rodents ranges from 25% to 40% (1). Antibody prevalence in dogs in Buenos Aires can be as high as 60%. Canine infection is mainly related to the presence of stagnant water and time spent outdoors (2).

Statistically, the most important sources of infection are leisure activities (31.4%); certain types of work, including garbage collection, sewer and construction work, and gardening (26.1%); and floods (16.1%) (3). During 2000–2001, a total of 93 cases were reported in this area. An outbreak that included 47 cases took place in March 2001, in Quilmes in the suburban area (Informe de Epidemiología de Quilmes, Buenos Aires, unpub. data). Four patients died with suspected leptospiral illness; three of these patients had laboratory-confirmed cases. We describe two cases with lethal pulmonary hemorrhage.

On July 2000 and March 2001, two women, ages 28 and 34, who lived in urban slum settlements, became ill. A high abundance of rodents inside their houses and in the neighborhood was reported in both cases. After 7–10 days of unspecific febrile illness, a severe pneumonia developed in both women. No jaundice, renal involvement, or thrombocytopenia was observed. When the patients were admitted to the critical-care unit, electrocardiograms were normal for both.

For one of the patients, empiric treatment was begun with 4 g of ceftriaxone plus 1 g of erythromycin daily. In the other, 800 mg/day of ciprofloxacin replaced the erythromycin. Endoscopic examination showed no lesions within the bronchial lumen, and abundant hemorrhagic secretions were obtained by aspiration. Both patients were mechanically ventilated and remained stable for the first 48 hours. Between the second and third day of ventilation, they became hypoxemic with acidosis and hypotension. Except for pulmonary hemorrhages, no other sign of bleeding was observed. Both patients died with cardiovascular collapse 10–11 days after onset of illness.

The microagglutination test with 10 serovars was positive for leptospirosis, as well as macroagglutination and enzyme-linked immunosorbent assay (ELISA) with leptospiral antigen, for immunoglobulin (Ig) M. Blood, urine, and bronchoalveolar lavage culture were negative for leptospira, as well as for other bacteria. IgM-capture ELISA (Andes serotype) for hantavirus was negative. Pathologic studies performed in one of the patients showed severe hemorrhage inside the pulmonary alveoli, with few interstitial lymphocytes; some septum tissue showed minimal enlargement. Warthin-Starry staining was negative for leptospira.

Rodents were captured near one patient’s house, and their kidneys were cultured in Ellinghousen-McCullough-Johnson-Harris medium. Three strains of *Leptospira interrogans* serovar *icterohaemorrhagiae* were isolated and characterized; laboratory guinea pigs were injected with the strains and several died 8–10 days later. Tegumentary jaundice was present, as well as abdominal hemorrhage foci. Pulmonary hemorrhages were observed bilaterally. Pericardial hemorrhages are remarkable as a possible cause of cardiopulmonary collapse. *Leptospira* were recovered from the liver and the kidneys, although brain and lung cultures were negative.

Another group of guinea pigs that had also been injected with *Leptospira* was humanely killed as soon as symptoms appeared. Necropsy showed primary lung injury. Lungs were pale with hemorrhages widely spread over the surface. Lesions were similar to those observed in one of the patients. Neither jaundice nor renal damage was found. *Leptospira* was isolated from kidneys, lungs, and brain. Jaundice has been reported in severe forms of human disease. Thrombocytopenia has been associated with renal failure and death in human patients.

Respiratory involvement in leptospirosis could be classified as a) mild to moderate (20% to 70% of patients), with pulmonary infiltrates commonly associated with jaundice and minimal alteration of renal function; b) severe, with jaundice, nephropathy, and hemorrhages (severe Weil's syndrome) (4); and c) fatal, with death occurring as a result of renal failure, myocarditis, or massive pulmonary hemorrhages with cardiovascular collapse.

In the past two decades, an increasing number of cases of leptospiral pulmonary hemorrhages have been reported, especially from Southeast Asia (5). In a review of leptospirosis in Brazil, death was associated with renal failure in 76.2% of fatal cases, while 3.5% were related to pulmonary hemorrhages (6). In the epidemic outbreak in Nicaragua in 1995, this form was considered the cause of death in the 40 fatal cases reported (7).

The two cases reported here were associated with pulmonary hemorrhage. This clinical form has not been previously reported in the Buenos Aires metropolitan area. Environmental and social factors, the prevalence of infection in reservoirs, and the virulence of the isolated strains must be considered in primary or critical-care units in the diagnosis of new cases, whether or not associated with an outbreak.
